# Postintravitreal Injection Endophthalmitis: Incidence, Characteristics, Management, and Outcome

**DOI:** 10.1155/2023/9212524

**Published:** 2023-11-06

**Authors:** Bar Davidov, Avi Ohayon, Omer Trivizki, Shulamit Schwartz, Shiri Shulman

**Affiliations:** ^1^Division of Ophthalmology, Tel Aviv Sourasky Medical Center, Tel Aviv, Israel; ^2^Affiliated to the Sackler School of Medicine, Tel Aviv University, Tel Aviv, Israel; ^3^Opthalmology Institute, Assuta Medical Centers, Tel Aviv, Israel; ^4^Affiliated to Faculty of Health Sciences, Ben-Gurion University of the Negev, Beersheba, Israel

## Abstract

**Purpose:**

Postintravitreal injection (IVI) endophthalmitis is a rare but devastating complication. Herein, we report the incidence ,and clinical and microbiological characteristics, as well as the visual outcome, in IVIs endophthalmitis in two medical centers.

**Methods:**

All patients undergoing intravitreal injections between 1/2018 and 12/2019 in two large medical centers were analyzed for post-IVI endophthalmitis.

**Results:**

Of the total of 51,356 IVIs performed, 23 cases of post-IVI endophthalmitis were diagnosed, yielding an overall incidence of 0.045%. The median interval from IVI to symptoms onset was 2 days (IQR: 1–5). Cultures were positive in 56% of the cases (100% Gram-positive bacteria and 76% coagulase-negative *staphylococcus*). Parameters associated with higher culture-positive rates included samples taken during vitrectomy, WBC on vitreous smear, the number of IVIs in the 12 months prior to presentation, and the time interval from last IVI to diagnostic sampling. At 6- and 12-month follow-up, the median change in VA (logMAR) was −1.10 (IQR: (−1.32)–(−0.40)) and −1.02 (IQR: (−1.10)–(−0.30)), respectively. Younger age and better BCVA at presentation were associated with better VA outcome, while positive culture result and systemic steroids treatment were each associated with the worse visual outcome. We found no difference in visual outcomes between PPV and TAI as a primary procedure.

**Conclusion:**

Post-IVI endophthalmitis is a rare complication, and most patients do not regain their initial VA. Certain parameters (clinical, microbiological, and therapeutic) may help anticipate the outcome and guide decision making regarding diagnosis and treatment.

## 1. Introduction

The use of intravitreal injections (IVIs) of steroidal and vascular endothelial growth factor (VEGF) antagonists has dramatically increased due to the widening spectrum of indications for its use, such as age-related macular degeneration (AMD), diabetic macular edema (DME), proliferative diabetic retinopathy (PDR), macular edema following retinal vein occlusion (RVO), and choroidal neovascularization (CNV) related to other diseases. Although highly effective, IVIs are not without risk, with infectious endophthalmitis being one of the more fearsome complications due to its poor prognosis. There is no consensus regarding the preinjection management to lower the risk of endophthalmitis. The American Academy of Ophthalmology recommends the application of a topical anesthetic, application of 5% or 10% povidone-iodine drops and/or periocular povidone-iodine eyelid preparation, insertion of a sterile speculum to separate the lids, and reapplication of povidone-iodine immediately over the injection site prior to injection. According to the literature, the incidence of endophthalmitis following a single IVI is as low as 0.056%; however, many of the indications for IVIs require multiple injections over a long period of time, ultimately leading to a higher overall cumulative risk [1].

Post-IVI endophthalmitis generally presents with redness, pain, decreased vision, and vitritis [2, 3]. Most commonly, patients present within 3 days of injection, with some presenting even several weeks after IVI [2, 3]. Management includes collecting vitreous sample using a vitreous tap or PPV, followed by injection of intravitreal antimicrobial agents. Systemic and topical antimicrobials and systemic, intravitreal, and topical steroids, as well as intravitreal silicone oil, are useful adjunctive therapies [4–6]. Cultures are positive in 52%–62% of the cases according to large series and meta-analyses described in the literature [7, 8]. Gram-positive bacteria cause >95% of the culture-positive cases, the most common being coagulase-negative staphylococci (*Staphylococcus epidermidis*: 60%–65%), followed by *Streptococcus* species (30% of the cases) [7, 9, 10]. While culture results impact on clinical management remains debatable, its value in visual outcome prognostication has been reported [4, 11–13].

Visual outcomes following post-IVI endophthalmitis vary considerably [3, 14]. Factors which may predict poor prognosis include positive culture results, with certain pathogens, namely, Streptococcus and Enterococcus species, portending the worst prognosis [3, 11, 15, 16].

The purpose of this study was to report the incidence, clinical and microbiological characteristics, visual outcome, and disease activity in post-IVI endophthalmitis and evaluate the relation of clinical and therapeutic factors to culture results and visual outcome. In addition, it was aimed to examine the effect of endophthalmitis on disease activity in the nAMD subgroup of post-IVI endophthalmitis patients.

## 2. Methods

This was a retrospective, case series of patients diagnosed with post-IVI endophthalmitis between January 2018 and December 2019 in two large medical centers. The Institutional Review Board approved the study protocol and waived patients' consent for it is a retrospective study. The research adhered to the tenets of the Declaration of Helsinki.

### 2.1. Inclusion and Exclusion Criteria

All eyes diagnosed with infectious endophthalmitis within 6 weeks of IVI of any agent with at least 12-month follow-up were included. Post-IVI endophthalmitis was defined as any case in which clinical suspicion was high enough to warrant surgical intervention with the means of vitreous tap and intravitreal antibiotics injection (“tap and inject,” TAI) and/or PPV. Exclusion criteria included patients treated for inflammatory endophthalmitis without TAI and/or PPV, endogenous endophthalmitis, intraocular surgery, or trauma more recent than last IVI, as well as cases with the follow-up period shorter than 12 months.

### 2.2. Injection Technique

The standardized method for IVI in both medical centers is as follows. All injections were performed at a designated clinic at a supine position. Sterile drape and lid speculum were used. Prior to injection, topical anesthetic drops were instilled followed by topical 5% povidone-iodine ophthalmic solution. Injection was performed with a 30-gauge needle, 3.5–4.0 mm from the limbus. Eye quadrant for needle insertion was chosen individually by the physician. A single dose of postinjection topical antibiotics was routinely instilled immediately postinjection. No additional prophylactic antibiotics are recommended at home.

Bevascizumab was dispensed in a syringe prepared by a compounding pharmacy, and ranibizumab was dispensed in a prefilled syringe. Aflibercept and triamcinolone were dispensed in a vial which was extracted to a syringe by the injecting physician immediately prior to IVI.

### 2.3. Management of Endophthalmitis

In both medical centers, all eyes with presumed infectious endophthalmitis underwent TAI or early PPV based on physician decision according to the clinical manifestation. Vitreous tap was conducted using a 25- or 27-gauge needle in order to aspirate vitreous and subsequently inject intravitreal antibiotics. Based on the physician decision and according to the clinical manifestation, in some cases, immediate PPV was conducted and IVI of antibiotics was performed. IVI of antibiotics in TAI or PPV includes vancomycin (1 mg/0.1 ml) and ceftazidime (2 mg/0.1 ml). In cases of penicillin allergy, amikacin (400 mg/0.1 ml) was injected as a substitute for ceftazidime. Culture results and sensitivities guided subsequent intravitreal antibiotics. Topical antibiotics drops were given to all the patients hourly around the clock and then tapered down based on clinical improvement. Based on the physician decision, fortified vancomycin (25 mg/ml) and fortified ceftazidime (50 mg/ml) or moxifloxacin hydrochloride (0.5%) were given. Cycloplegia (topical atropine sulfate 0.5% drops or cyclopentolate 2% drops) was given in all cases. Topical steroids (dexamethasone 0.1% or prednisolone acetate 1%) were also given in all cases. Systemic steroids were added according to physician discretion, given daily (0.5–1 mg/kg/day) and tapered gradually according to clinical response over a 6–8 week period. Patients were evaluated daily, and upon clinical improvement, topical treatment was tapered down and follow-up intervals were extended.

### 2.4. Data Collected

The yearly number of IVIs was determined using billing codes. Medical records were reviewed to identify patients treated for endophthalmitis within 6 weeks after IVI during the study period. Collected data included demographics, underlying diagnosis indicating IVI treatment, treatment history (agent injected, number of injections at 12 months previous to endophthalmitis, and date of last IVI), clinical findings on slit-lamp biomicroscopy at presentation of endophthalmitis (corneal edema, anterior chamber cells, anterior chamber fibrin, hypopyon, posterior synechiae, vitritis, preretinal exudates, and intraretinal hemorrhages), initial procedure (TAI or PPV), secondary procedure (PPV), topical antibiotics drops treatment, systemic steroids treatment, white blood cells (WBCs) on direct smear, culture results, last recorded best corrected visual acuity (BCVA) before IVI (“baseline BCVA”), and BCVA at presentation and at 6- and 12-month follow-up. BCVA was measured using Snellen charts and subsequently converted into logMAR values for statistical analysis. Time intervals between last IVI and symptoms onset, between IVI and the first procedure, and between symptoms onset and the first procedure were recorded.

### 2.5. Outcomes

Primary outcome was BCVA at 6 and 12 months following treatment as well as the change in BCVA at these time points in comparison to the baseline BCVA and BCVA at presentation. Secondary outcomes were the incidence of post-IVI endophthalmitis, BCVA, and clinical findings on presentation, as well as microbiological characteristics.

In addition, we evaluated the association of several demographic, clinical, laboratory, and management factors to visual outcomes including return to the baseline BCVA, any improvement in BCVA from presentation, and relatively low BCVA (defined as BCVA of 6/60 and lower).

### 2.6. Statistical Analysis

Categorical variables were summarized as the frequency and percentage. Continuous variables were evaluated for normal distribution using histogram and reported as the median and the interquartile range (IQR). The chi-square test and Fishers' exact test were applied to compare proportions between categorical variables. Continuous variables were compared between categories using the Kruskal–Wallis test and the Mann–Whitney test. The Spearman correlation coefficient was used to study the association between continuous variables. The Wilcoxon test was used to compare continuous variables between 2 time points. All statistical tests were two sided, and *P* < 0.05 was considered statistically significant. Data were analyzed using IBM SPSS statistical software version 25.0 (Armonk, NY: IBM Corp).

## 3. Results

### 3.1. Endophthalmitis Rates

During the study period, a total of 51,356 IVIs were performed in both medical centers. The injections rate of the different medications is detailed in Table 1.

23 cases of post-IVI endophthalmitis were diagnosed in 23 patients; of them, 11 (48%) were following aflibercept injection, 10 (43%) following bevacizumab injection, 1 (4%) following triamcinolone acetonide injection, and 1 (4%) following dexamethasone intravitreal implant, giving an overall incidence of 0.045% (0.041% following anti-VEGF agents IVIs and 0.364% following steroidal agents IVIs). No endophthalmitis cases were seen after ranibizumab injections. The higher incidence of endophthalmitis was found following steroidal agents' injections in comparison to anti-VEGF agents' injections (3.64/1,000 patients vs. 0.41/1,000 patients, *P* = 0.025). The higher incidence of endophthalmitis was found following bevacizumab (0.045/1000 patients) and aflibercept (0.063/1000 patients) injections in comparison to the endophthalmitis rate following ranibizumab injections (0/1000 patients, *P* = 0.37 and *P* = 0.09, respectively) but not following bevacizumab injections in comparison to aflibercept injections (*P* = 0.437, Table 1).

### 3.2. Demographics

Of the 23 cases (16 (70%) female, 7 (30%) male, and median age 74), 14 (61%) cases involved the left eye and 9 (39%) cases of the right eye. The most common indication for IVI was nAMD in 16 (70%) patients, followed by diabetic macular edema (DME) and vein occlusion, each in 2 (9%) patients, and pseudophakic cystoid macular edema (PCME), multifocal choroiditis, and choroidal rupture-related CNV, each in one (4%) patient (Table 2). The median number of injections in the 12 months previous to endophthalmitis was 8 intravitreal injections (IQR 5.75–10.00).

### 3.3. Clinical Presentation

The most common presenting symptom was decrease in vision in 20 (87%) patients, and the most common clinical signs were anterior chamber cells and vitritis seen in all patients (100%). The incidence of presenting signs and symptoms is presented in Table 2. The median interval from IVI to symptoms onset was 2 days (IQR: 1–5), from symptoms onset to the first procedure was 1 day (IQR: 0–3), and from IVI to the first procedure was 4 days (IQR: 2–7) (Table 2).

### 3.4. Management

Topical antibiotics drops were initiated at admission in all 23 (100%) patients, of which 20 (87%) received fortified vancomycin (25 mg/ml) and fortified ceftazidime (50 mg/ml) and 3 (13%) received moxifloxacin hydrochloride (0.5%). The first procedure performed was early PPV in 12 (52%) patients and TAI in 11 (48%) patients, at which all patients received IVI of vancomycin (1 mg/0.1 ml) and ceftazidime (2 mg/0.1 ml), and a vitreous sample was collected. Two (9%) patients underwent PPV as a second procedure, of which one (50%) was after TAI and one (50%) after early PPV. One (4%) patient received additional IVIs of vancomycin (1 mg/0.1 ml) 5 days after TAI. In 11 (48%) patients, systemic steroids were added (Table 2). Time intervals from IVI to symptoms onset and to first performed procedure are detailed in Table 2.

### 3.5. Laboratory Results

Direct smear of the vitreous sample (from TAI or PPV) showed white blood cells (WBCs) in 12 (52%) patients, of which 11/12 (92%) eventually had positive culture results (positive predictive value of 92%). WBC on direct smear were associated with a higher rate of positive culture result (91.7% vs. 18.2%, *P* = 0.001).

Cultures of vitreous samples were positive in 13 (56%) cases and negative in 7 (30%) cases and in 3 (13%) cases, vitreous sample volume, collected by TAI procedure, was insufficient for laboratory evaluation (Table 2). Of the culture-positive cases, 9 vitreous samples were collected by vitrectomy and 4 by TAI, resulting in a culture-positive rate of 75% (9/12 patients) in early PPV compared to 36% (4/11 patients) in TAI. PPV (as early or late procedure) had a higher culture-positive rate compared to TAI only cases (76.9% vs. 30.0%, *P* = 0.04).

All pathogens (100%) were Gram-positive bacteria, most commonly coagulase-negative staphylococcus (CoNS) (11 cases, 76.2%) as detailed in Table 2.

A higher culture-positive rate was found in cases with longer time interval from IVI to any first procedure (median 7, IQR: 4–10 vs. median 3.5, IQR: 2–4, *P* = 0.007). Other parameters analyzed were not associated with a higher culture-positive rate (*P* > 0.05). These include patients' age, injected agent, presenting signs or symptoms, BCVA at presentation or its decrease from baseline, time interval from IVI to symptoms onset, and time interval from symptoms onset to the first procedure.

### 3.6. Visual Outcome

Median BCVA at the baseline, at presentation, and at 6- and 12-month follow-up is detailed in Table 3.

**Figure 1:**
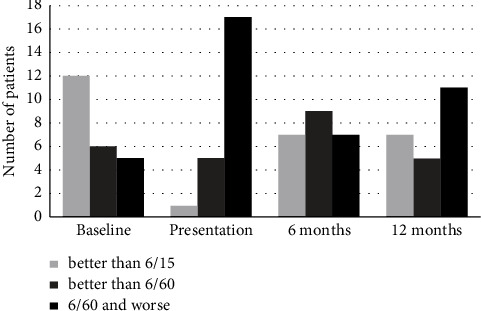
BCVA distribution along the study period.

20 (87%) patients presented with vision loss in comparison to the baseline BCVA. BCVA improvement from presentation was documented in 18 (78%) and 17 (74%) patients at 6- and 12-month follow-up, respectively. 8 (35%) and 9 (39%) patients return to their baseline vision (within one line from the baseline BCVA) at 6- and 12-month follow-up, respectively. BCVA distribution along the study period is shown in Figure 1.

Of the demographic parameters, younger age was associated with the higher rate of return to the baseline BCVA (median 71, IQR: 63–73.5 years vs. median 78, IQR: 73.5–85, *p* = 0.03).

Of the presenting signs and symptoms, better BCVA on presentation was associated, with higher rates of return to the baseline BCVA (median 1, IQR: 0.55–1.67 vs. median 2.1, IQR: 1.47–2.25, *P* = 0.013).

Of the laboratory findings, positive culture results were associated with worse visual outcome (BCVA of 6/60 and worse, 53.9% vs. 0%, *P* = 0.017).

There was no difference in final VA between the two primary procedures (early PPV or TAI) (*P* > 0.999). Analysis of baseline characteristics revealed no statistically significant difference between the two groups (early PPV vs. TAI), including demographics, disease characteristics, presenting signs and symptoms, laboratory findings, systemic steroids use and time intervals between injection, symptoms onset, and primary procedure.

Patients who received systemic steroids treatment were found to have a lower rate of improvement in VA along the follow-up (54.6% vs. 100% *P* = 0.035). Analysis of baseline characteristics revealed no statistically significant difference between the two groups (systemic steroids treatment vs. no systemic steroids treatment), including demographics, disease characteristics, presenting signs and symptoms, laboratory findings, initial procedure (TAI vs. early PPV) and time intervals between injection, symptoms onset, and primary procedure.

## 4. Discussion

In this retrospective, case-series study, we report the incidence rate, clinical and microbiological characteristics, management, and outcome of post-IVI endophthalmitis in two medical centers, as well as evaluate the correlation between the presenting signs and symptoms, culture results, management, and visual outcome.

### 4.1. Incidence

In our study, the overall incidence of post-IVI endophthalmitis was 0.045% which is comparable to the incidence reported in the literature [1]. The incidence of endophthalmitis after steroidal agents' injection was significantly higher in comparison to anti-VEGF agents (Table 1) as previously described [4].

Bevacizumab and aflibercept each showed significantly higher rates of endophthalmitis compared to ranibizumab (Table 1). This finding is possibly due to the use of prefilled syringe of ranibizumab. Mun et al. reported a significant association of bevacizumab with endophthalmitis compared to nonbevacizumab drugs, combining ranibizumab and aflibercept [17]. A similar finding was reported by Xu et al. in a study of 258, 357 anti-VEGF IVIs [18]. This finding might be related to the division process into individual aliquots in bevacizumab vials. A higher incidence of post-aflibercept endophthalmitis compared to ranibizumab was described before by Kiss et al. [19].

### 4.2. Presentation

The most common presenting symptom was decrease in vision, followed by pain and redness, as described before in the literature [14]. The median interval between IVI and symptoms onset was 2 (IQR 1–5), which is comparable to the incidences reported in the literature [18, 20].

### 4.3. Laboratory Results

WBCs on direct smear of vitreous sample were associated with a higher rate of positive culture results. This finding may assist in early prognostication as positive culture results were shown to have worse visual outcome [4, 11–13]. The overall culture-positive rate (57%) was comparable to the rate described in the literature with a higher culture-positive rate among samples taken during vitrectomy (75%) versus vitreous tap (36%) [7, 8]. This finding might represent a higher yield of a vitreous sample taken by vitrectomy in comparison to vitreous tap. It was suggested to be a result of selection bias of cases that are more severe clinically, with higher bacterial load in the vitreous taken to early vitrectomy [18]. On the other hand, in our series, the early PPV and TAI subgroups did not differ in several presenting signs and symptoms as detailed. In 3 cases, the vitreous sample volume was insufficient for laboratory evaluation. Although this finding represents real life results, it might as well bias our results.

In one case, there was a positive culture of *Globicatella sanguinis* which grew as a coinfection with *Staphylococcus simulans*. In this case, visual acuity deteriorated from 6/10 preinjection to hand motion 12 months following the event of endophthalmitis. To our knowledge, we report the first post-IVI endophthalmitis caused by *Globicatella sanguinis*, an uncommon cause of human infection that affects the bloodstream, urinary tract, and central nervous system [21–24]. Only one case of *Globicatella sanguinis* endophthalmitis was described in the literature of a 9-year-old healthy boy presented with corneal abscess and endophthalmitis [25]. *Globicatella sanguinis* is a catalase-negative, nonhemolytic, Gram-positive coccus which was first described in 1992 by Collins and colleagues [26]. This microorganism has been noted as a colonizer of the skin, which might explain the coinfection with *Staphylococcus simulans*, a member of the CoNS group, known to colonize the skin and mucous membranes [24, 27]. We believe that the low incidence described in the literature is due to relatively recent identification of *Globicatella sanguinis* as a specific pathogen, as well as advanced laboratory tests required for its identification, as standard phenotypic techniques are usually insufficient [25].

The time interval from IVI to the first procedure was significantly associated with higher culture-positive rates. This interval may allow a longer time for bacterial growth and thus a higher bacterial load, resulting in a higher rate of positive culture results.

In a series of 65 cases of endophthalmitis post-IVI, Dossarps et al. reported no significant difference in the median number of IVIs before endophthalmitis between culture-positive and culture-negative cases [28]. Similarly, we found that accumulative IVIs number at 12 months before endophthalmitis was not associated with a higher culture-positive rate.

Evidence regarding the correlation between presenting signs and symptoms to culture results in endophthalmitis is inconsistent. While Chirag et al. found no significant correlation, Collins et al. reported a correlation between several parameters of the clinical presentation and microbiologic culture results [26, 29]. In our study, presenting signs and symptoms, BCVA at presentation or its decrease from the baseline, were not associated with a higher rate of positive culture results. These findings might be due to the narrow spectrum of culture results in our study (100% were Gram-positive bacteria and 76% were CoNS positive). Alternatively, it might be due to the relatively small number of endophthalmitis cases in our study.

### 4.4. Visual Outcome

The rate of vision improvement (within one line from the baseline BCVA) compared to presentation was 78% and 74% at 6- and 12-month follow-up, respectively. 35% and 39% of the patients return to their baseline vision at the 6- and 12-month follow-up, respectively. These rates are comparable to those described in other studies [18].

Of the demographic parameters evaluated, younger age was associated with better visual outcome. In a recent study by Xu et al. of 40 patients with post anti-VEGF IVI endophthalmitis, younger age was associated with better visual outcome as well [18].

Of the presenting symptoms, we found that better visual acuity at presentation was associated with the better visual outcome, while Xu et al. reported that BCVA at presentation did not correlate to BCVA at the 6-month follow-up [18].

A positive culture result was significantly associated with the worse visual outcome, a finding that is inconsistent in previous studies and seems to be affected by the specific pathogens among the culture-positive subgroup [1, 18, 28, 30].

We found no difference in visual outcomes between PPV and TAI as a primary procedure. In contrast to endophthalmitis following cataract extraction surgery, there are no guidelines for the treatment of post-IVI endophthalmitis [31]. Based on the retrospective analyses published, there seems to be no significant difference in the outcome between patients treated first by TAI or PPV and our study supports this finding [32].

We found worse visual outcome in patients who received systemic steroids treatment. There is no consensus in the literature regarding the use of systemic corticosteroids in endophthalmitis. A retrospective trial by Robbins et al. of 133 eyes with endophthalmitis (23 post-IVI) found a higher rate of vision improvement of 3 lines or more among the 25% of patients treated with systemic steroids [33]. They reported that oral steroids use was associated with culture-positive endophthalmitis, hypotony, conjunctival hyperemia, and anterior chamber fibrin on examination. We found a 48% rate of oral steroids treatment, and our analysis revealed no difference in demographic characteristics, disease characteristics, presenting signs and symptoms, culture results, primary procedure, and measured time intervals between patients treated with systemic steroids and patients that were not.

Our study has several limitations. First, its retrospective nature weakens the conclusions, and the relatively small number of endophthalmitis cases did not allow a multivariable analysis. Although the retrospective nature of this study allows an underestimation of the endophthalmitis rate, we believe the possibility of missing reports is low as our centers are of the largest in the country and the communication between retina teams in a small country like ours is fluent. Second, although all patients were managed by one retina team, the chosen primary procedure and the use of systemic steroids were chosen according to physicians' decision and not by defined protocols, as the literature lacks such protocols, which may result in selection bias.

## 5. Conclusion

We report an overall endophthalmitis rate of 0.045% after IVI of anti-VEGF or corticosteroids agents in two large medical centers. This rate was significantly higher in steroidal agents (vs. anti-VEGF agents) and in bevacizumab or aflibercept (vs. ranibizumab). Younger age and better VA at presentation were associated with the better visual outcome, while positive culture result and systemic steroids treatment were each associated with the worse visual outcome. The chosen initial procedure by TAI versus PPV was not associated with a different visual outcome.

Our findings may help anticipate outcome and guide decision making regarding diagnosis and treatment of postinjection endophthalmitis. Further prospective trials are warranted in order to establish management guidelines in post-IVI endophthalmitis.

## Figures and Tables

**Table 1:** Intravitreal injections rate and the endophthalmitis rate in two medical centers during the study period.

Agents	Anti-VEGF agents				Steroidal agents			Total
	Bevacizumab	Ranibizumab	Aflibercept	All	Triamcinolone acetonide injectable suspension	Dexamethasone intravitreal implant	All	
Number of injections	22397	10840	17570	50807	47	502	549	51356
Relative injection rate	43.6%	21.1%	34.2%	98.9%	0.1%	1.0%	1.1%	100.0%
Endophthalmitis events	10	0	11	21	1	1	2	23
Endophthalmitis rate	0.045%^*∗*^	0.000%^*∗*^	0.063%^*∗*^	0.041%^*∗*^	2.128%^*∗*^	0.199%^*∗*^	0.364%^*∗*^	0.045%^*∗*^

(^*∗*^) *P* = 0.025 for steroidal agents in comparison to anti-VEGF agents; *P* = 0.037 for bevacizumab in comparison to ranibizumab; *P* = 0.09 for aflibercept in comparison to ranibizumab; *P* = 0.437 for aflibercept in comparison to bevacizumab; *P* = 0.164 for triamcinolone acetonide injectable suspension in comparison to dexamethasone intravitreal implant.

**Table 2:** Demographics, clinical presentation, laboratory findings, and management of 23 post-IVI endophthalmitis cases.

Sex	
Female (%)	16/23 (70)
Male (%)	7/23 (30)
Age, median, years (IQR)	74.0 (70–83)
Indication for IVI	
Age-related macular degeneration (%)	16/23 (70)
Diabetic macular edema (%)	2/23 (9)
Retinal vein occlusion (%)	2/23 (9)
Pseudophakic cystoid macular edema (%)	1/23 (4)
Multifocal choroiditis (%)	1/23 (4)
Choroidal rupture-related CNV (%)	1/23 (4)
Clinical presentation	
Symptoms	
Decrease in vision (%)	20/23 (87)
Pain (%)	16/23 (70)
Redness (%)	12/23 (52)
Signs	
Corneal edema (%)	8/23 (35)
Anterior chamber cells (%)	23/23 (100)
Fibrin (%)	8/23 (35)
Hypopyon (%)	9/23 (39)
Posterior synechiae (%)	2/23 (9)
Intraretinal hemorrhages (%)	8/23 (35)
Preretinal exudates (%)	8/23 (35)
Vitritis (%)	23/23 (100)
IOP (mmHg), median (IQR)	14 (10–18)
Time intervals	
Injection: symptoms onset interval, median, days (IQR)	2 (1–5)
Symptoms onset: 1st procedure interval, median, days (IQR)	1 (0–3)
Injection: 1st procedure interval, median, days (IQR)	4 (2–7)
Laboratory findings	
WBC on direct smear	
Yes (%)	12/23 (52)
No (%)	11/23 (48)
Culture	
Positive (%)	13/23 (57)
PPV sample (%)	10/13 (77)
TAI sample (%)	3/13 (23)
Negative (%)	7/23 (30)
Insufficient sample volume (%)	3/23 (13)
Pathogen (%)	
Coagulase-negative staphylococci (CoNS) (%)	11/13 (85)
*Staphylococcus epidermidis* (%)	10/13 (77)
*Staphylococcus simulans* (%)	1/13 (8)
*Staphylococcus aureus* (%)	1/13 (8)
*Streptococcus sanguinis* (%)	1/13 (8)
*Globicatella sanguinis* (%)	1/13 (8)
Management	
Systemic steroids	
Yes (%)	11/23 (48)
No (%)	12/23 (52)
Primary procedure	
TAI (%)	11/23 (48)
PPV (%)	12/23 (52)
Second procedure (%)	2/23 (9)
PPV after TAI (%)	1/2 (50)
Seconds PPV (%)	1/2 (50)

CNV: choroidal neovascularization; IOP: intraocular pressure; IQR: interquartile range; IVI: intravitreal injection; PPV: pars plana vitrectomy; TAI: tap and inject. WBC: white blood cells.

**Table 3:** Best corrected visual acuity (logMAR) in post-IVI endophthalmitis along the study period.

	BCVA (logMAR)	BCVA (Snellen)
Baseline, median (IQR)	0.30 (0.18–0.70)	≈6/12
At presentation, median (IQR)	1.78 (0.82–2.10)	≈6/120-CF
Change from the baseline	+1.26 ([+0.40]–[+1.68])	
At 6 m follow-up, median (IQR)	0.61 (0.30–1.07)	≈6/24
Change from the baseline	+0.19 ([0.00]–[+0.44])	
Change from presentation	−0.88 ([−1.30]–[−0.20])	
At 12 m follow-up, median (IQR)	0.74 (0.27–1.32)	≈6/24
Change from the baseline	+0.19 ([0.00]–[+0.62])	
Change from presentation	−0.92 ([−1.18]–[−0.05])	

IQR: interquartile range; IVI: intravitreal injection; logMAR: logarithm of the minimum angle of resolution.

## Data Availability

The data used to support the findings of this study are included within the article.
